# Notes and News

**Published:** 1898-05-21

**Authors:** 


### NOTES AND NEWS.

(COLLECTED AND COMMUNICATED.)

HOSPITAL MEETINGS.

Hospital for Sick Children, Great Ormond Street.—Annual Court, Wednesday, May 25th, 4.30 p.m., at the Hospital.

THE Duchess of Teck Memorial Fund now amounts to upwards of £9,000.

THE seventeenth congress of the Sanitary Institute is to be held on September 27th at Birmingham, under the presidency of Sir Joseph Fayrer.

AT the festival dinner of the City of London Hospital, in celebration of the fiftieth anniversary, donations and subscriptions amounted to £2,866.

MESSRS. KELLY AND CO., of handbook fame, have offered to direct, free of charge, 35,000 envelopes in connection with the Prince of Wales's Hospital Fund.

THE City of London Truss Society has just completed its ninety-first year of existence, and has relieved upwards of half a million sufferers.

IN the recent railway ambulance competition the silver challenge shield presented by the St. John Ambulance Association and the first prize were awarded to the Great Eastern corps, this being the second year this team has held the shield; the Great Northern took second prize, while the third fell to the North-Eastern.

THE boys of Christ's Hospital have generously contributed £15 to the funds of the *Arethusa* and *Chichester* training ships, Greenhithe, Kent, this being their twenty-seventh annual subscription of that amount towards helping poor boys of good character who wish to go to sea.

THE Spectaclemakers' Company, of which the Lord Mayor is this year master, purposes to hold an optical exhibition. Sir Reginald Hanson, M.P., has promised a substantial donation, on condition that the most noteworthy exhibits are afterwards sent on to the Paris Exhibition in 1900.

THE Marquis of Dufferin and Ava, in issuing an appeal for the Royal Free Hospital, and asking the public to support the festival dinner at the Hotel Cecil on May 20th, calls special attention to the valuable work done by medical women in India, many of whom are past students of the Royal Free.

A SCHEME is before the Emperor of Austria for improving the health of Vienna by establishing a large sanitary ring of open spaces round the city to act as the necessary lungs, these being conspicuously few in this beautiful city.

WE understand that the Bengal Government have applied to the Government of Bombay for the services of twenty-five medical men and as many nurses who have been employed on plague duty.

CONSIDERABLE dissatisfaction is being expressed in India at the manner in which the rewards for plague service are being given. It is stated that these allowances are only being paid in full to those officers who were transferred to plague work from purely military duties.

MANY improvements are rife in Ceylon, so far as hospital accommodation is concerned. Recently the Brodie Memorial Ward, a surgical ward for women and children, has been added to the Lady Havelock Hospital, while a new seamen's ward, now being erected in grounds adjoining the civil hospital, will soon be ready for occupation, and will accommodate 24 patients. The old seamen's ward is to be turned into a pauper's ward, additional beds for such patients being badly needed.

THE Newsvendors' Benevolent and Provident Institution was fortunate in its chairman, Mr. C. B. Harmsworth, at its annual dinner held on the 11th inst. The dinner took place at the Hotel Cecil, the attendance was large and representative, the speaking was exceptionally good, and the quality of the music was unusually high, for Madame Antoinette Stirling was good enough to give her services for the entertainment of the guests. Few men earn their living harder than newsvendors, and we are gratified to know that the dinner resulted in a record collection, and that upwards of £350 was contributed in annual subscriptions. This institution has been in existence for sixty years, and its vitality may be realised from the circumstance that the managers are still able to secure the support and influence of the proprietors of both the oldest and the youngest of the great metropolitan daily papers.

SOME of the provincial papers have worked themselves up into a state of feverish excitement as to the treatment of prisoners in English prisons, and are endeavouring to inflame and prejudice the mind of the public. For instance, to speak of "starvation" and "brutal suffering" in connection with the dietary of English prisons is simply ridiculous. Possibly some of the prisoners, though not all, might like to have more food, but they have enough for health and to enable them to do their work. Prisoners have the right of free access to the doctor, and they are not slow to take advantage of this privilege. If a prisoner dies, the nature and duration of his illness are closely investigated by the coroner at the time of the holding of the inquest. Justice demands that we shall not convert our prisons into palaces, and the present outcry for luxurious treatment and over-feeding is as ill-judged as it is unjust to the honest poor who have to maintain a hard struggle for existence.

